# Maleic Anhydride and Its Derivatives: A Brief Review of Reactivity and Properties in Radical (Co)Polymerizations

**DOI:** 10.1002/marc.202500551

**Published:** 2025-10-08

**Authors:** Michael‐Phillip Smith, Bert Klumperman

**Affiliations:** ^1^ Department of Chemistry and Polymer Science Stellenbosch University Stellenbosch South Africa

**Keywords:** controlled radical polymerization, conventional polymerization, copolymerization, maleic anhydride, polymerization

## Abstract

Maleic anhydride (MAnh) and its derivatives comprise a collection of underutilized monomer classes, each with unique reactivities and properties, which afford the design and synthesis of highly functional (co)polymers. This review explores the opportunities and limitations associated with maleic anhydride and its derivatives in conventional radical and controlled radical polymerization techniques. The potential of these monomers to create (co)polymers with desirable properties in advanced polymer design is highlighted.

## Graphical abstract

Maleic anhydride (MAnh) and its derivatives are underutilized monomer classes, each with unique reactivities and properties, which afford the synthesis of highly functional (co)polymers. This review explores the current understanding, opportunities, and limitations associated with maleic anhydride and its derivatives in conventional radical and controlled radical polymerization techniques.

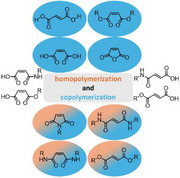

## 1 Introduction

The advancement of polymer science over recent years has created a surge in the complexity of technological applications, demanding more intentionally engineered polymers. Applications such as controlled drug release delivery systems [1, 2], hydrogels [3, 4], and functional surface tethering [5, 6] require bespoke polymers that have desirable properties, e.g., amphiphilicity, functional handles, and stimulus‐responsiveness. To achieve both the amphiphilicity and functionality required for applications, pre‐ or post‐polymerization modification of a (co)polymer system is essential. Post‐polymerization can, for example, be achieved via the esterification of acrylic acid‐containing polymers [7]. Depending on the degree of modification, a balance can be struck between amphiphilicity and functionality; however, this approach does not promise quantitative conversion and is functional group sensitive, which may result in batch‐to‐batch variation [7]. Alternatively, pre‐polymerization functionalization allows for the preparation of bespoke monomers [8]. Lastly, it is possible to compromise and instead utilize the library of acrylates or acrylamides available from commercial sources. Although there is work on activated ester variants of acrylic acid and methacrylic acid that can be (co)polymerized and easily modified in a post‐polymerization process [9, 10, 11], there is a need for further research into monomers that can be easily modified to afford different chemical functionalities for more unique polymer systems with required amphiphilicity and functionality.

Maleic anhydride (MAnh) and its derivatives have been utilized less in radical (co)polymerizations in comparison to acrylates (<1000 vs >500 000, respectively, publications on Scifinder*^n^*, 12‐12‐2024, Figure 1). The significant difference highlights the notable under‐utilization of these monomer classes in radical polymerization. We suspect that the monomer classes are underutilized because of limited knowledge and confidence with implementation within the community in comparison to acrylates and methacrylates. We hope that through this review, the community will become more familiar with these monomer classes and note their potential for future applications. In this work, each monomer class will be reintroduced, and the current radical‐mediated (co)polymerization techniques will be listed and outlined, including homopolymerization and copolymerization via conventional radical and controlled radical polymerization techniques.

**FIGURE 1:**
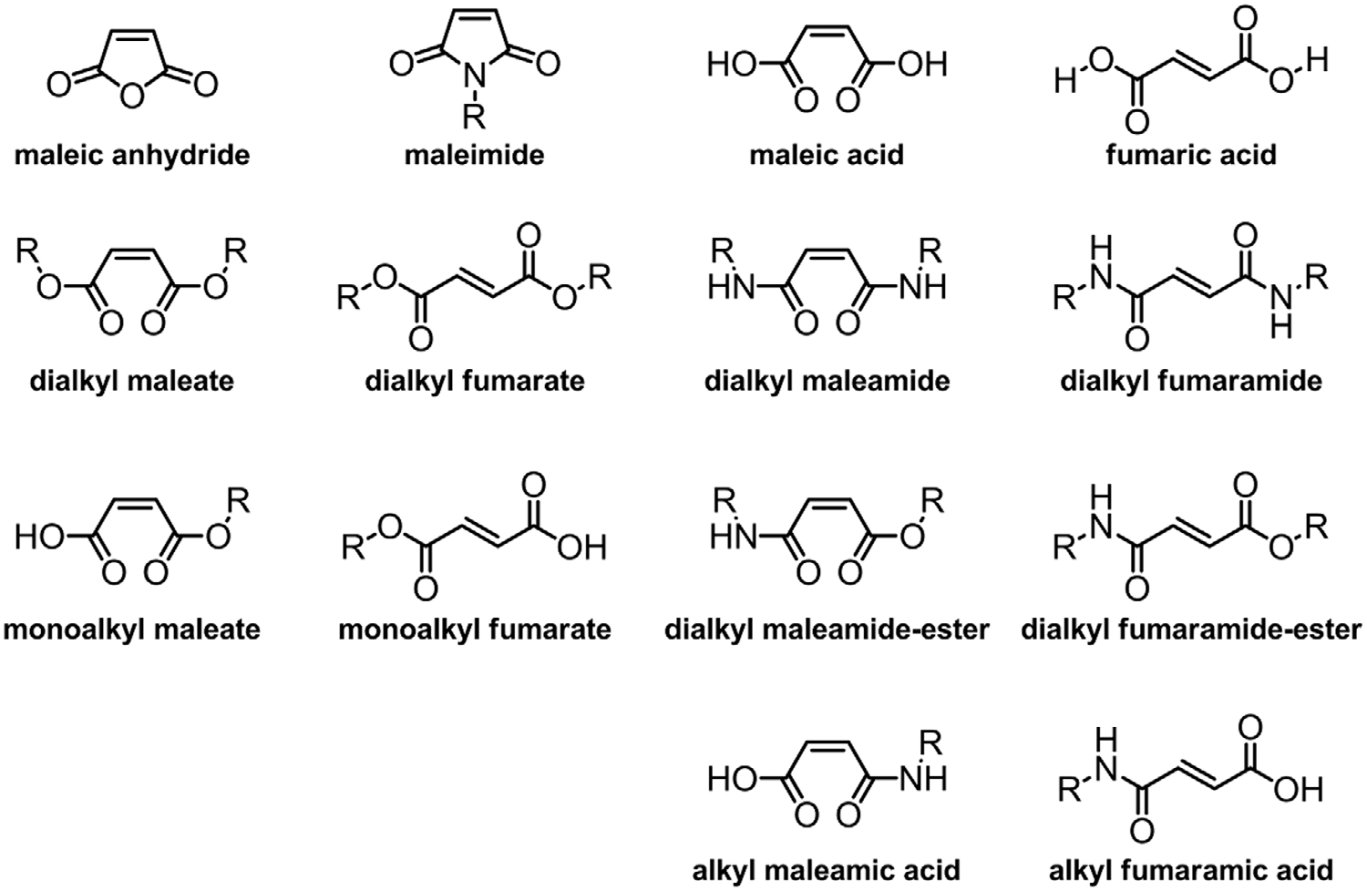
Chemical structures of maleic anhydride and its derivatives.

## 2 Maleic Anhydride

**FIGURE 2:**
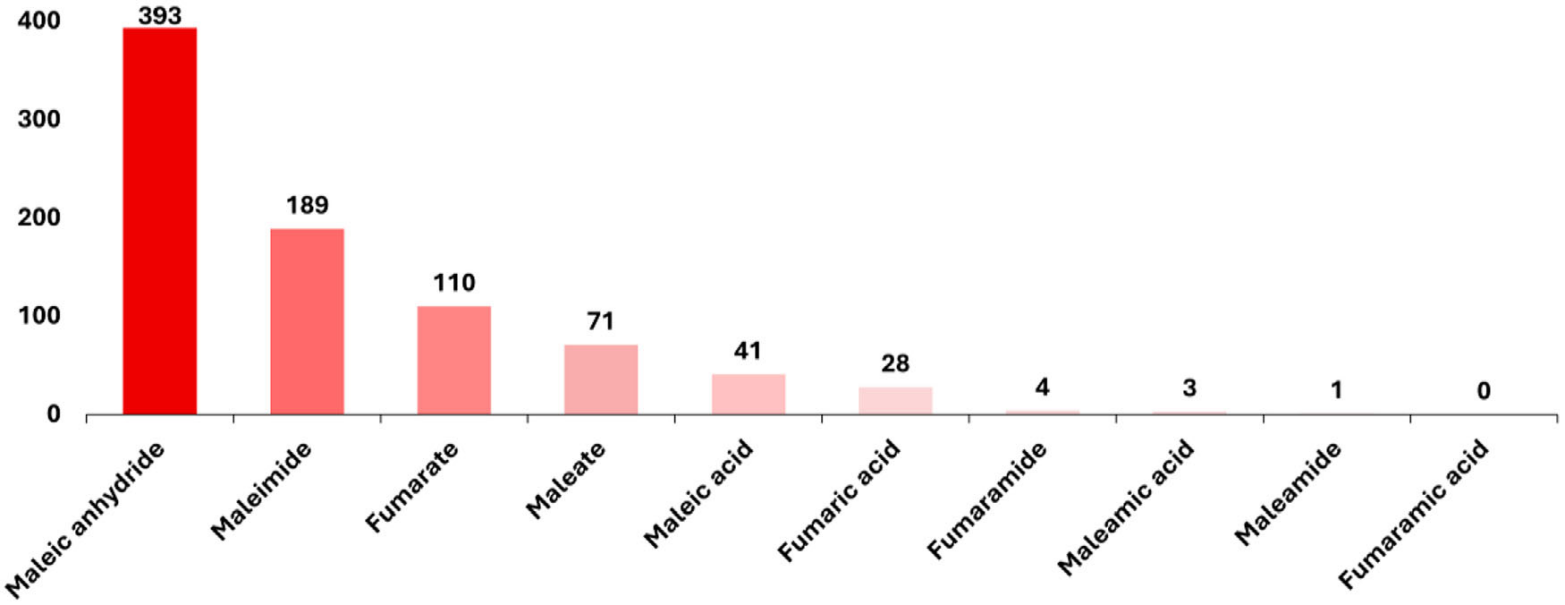
Number of publications per monomer class, search conducted on Scifinder^n^ (12‐12‐2024) with searches based on “Monomer name” and “title‐radical polymerization” or “title‐radical copolymerization”. Numbers were determined by the number of reviews and articles.

Among its derivatives, MAnh is the most studied, 47% (393 publications), of the derivatives (Figure 2). However, it is still neglected in comparison to other monomers utilized in radical polymerizations, such as methyl acrylate, butyl acrylate, and methyl methacrylate. MAnh is a cyclic anhydride‐containing monomer, with an alkene moiety conjugated to the anhydride functionality (Figure 1). It can be produced from both renewable (*n*‐butanol and furfural) [12] and non‐renewable (benzene, propylene, and chlorotoluene) feedstocks [13]. The unique conjugation pattern results in an electron‐deficient (acceptor) alkene, which readily undergoes an alternating copolymerization with electron‐rich (donor) monomers such as styrene [14, 15]. The anhydride functionality affords alkene hydrogen atoms that are highly susceptible to chain transfer reactions in radical polymerizations, resulting in lower molecular weight polymers in certain scenarios [16]. One of MAnh's most noteworthy features is its ease of modification, where the anhydride functionality lends itself to facile modification via ring‐opening reactions [17, 18]. The modifications can be readily conducted with either primary amines, secondary amines [19, 20], or alcohols [21]. MAnh can be modified by various means to afford other monomeric derivatives.

### 2.1 Conventional Radical Polymerization

MAnh does not undergo radical homopolymerization, except under extreme conditions to afford the generation of oligomeric chains (dimers to pentamers) [18, 22]. MAnh instead partakes in copolymerizations with electron‐rich comonomers [23], to afford copolymers that have a tendency toward alternating comonomer insertion. The electron‐deficient nature of the vinyl bond of MAnh proves helpful in copolymerizations with stable electron‐rich comonomers that do not readily homopolymerize, such as furans and aromatic compounds like phenanthrene and stilbenes [24, 25, 26, 27]. The incorporation of MAnh in some instances of copolymerization can further increase the rate of polymerization due to the acceptor (electron‐deficient vinyl bond) nature of the monomer, complimenting the donor (electron‐rich vinyl bond) monomers. This is exemplified by comparing the copolymerization of styrene and maleic anhydride to that of the homopolymerization of styrene [23].

The use of MAnh in a copolymerization reaction leads to a copolymer with regularly spaced functional handles for post‐polymerization functionalization [13] and imparts crosslinking ability [13, 18]. Some monomers that have been copolymerized via conventional radical copolymerization with MAnh include methacrylates [28, 29], methyl methacrylate [30], styrenics [31, 32, 33, 34, 35, 36, 37, 38, 39, 40, 41], olefins [24, 34, 42, 43, 44, 45], vinyl ethers [24, 46, 47, 48, 49, 50, 51, 52, 53], furans [24], vinyl acetate [54, 55, 56], phenylethyne [57], stilbenes [26, 27, 58], acrylamides [59, 60], *N*‐vinylpyrrolidone [61], acrylonitrile [62], dienes [63, 64, 65], norbornene [14, 66, 67], and aromatic compounds [25, 68, 69]. MAnh is copolymerized under conventional radical conditions at temperatures ranging from 60°C to 140°C [41], where the majority of copolymerizations utilize 70°C. It is recommended that the reaction temperature be kept below 200°C to limit decarboxylation of the anhydride moiety on the monomer [70].

**FIGURE 3:**
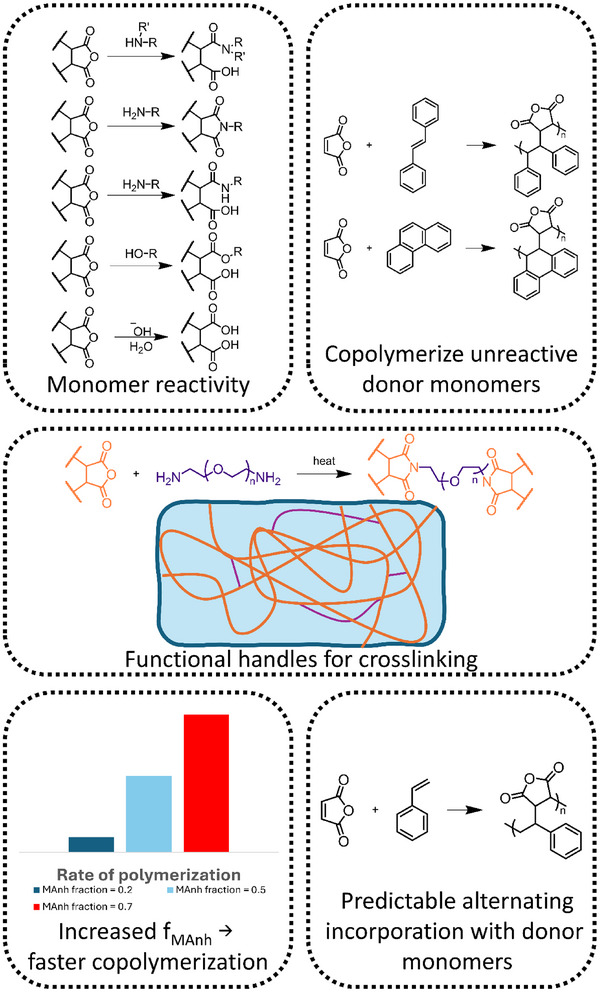
The versatility of maleic anhydride as a functional monomer.

MAnh is a predictable comonomer with a tendency toward an alternating comonomer incorporation when copolymerized with donor monomers. This donor‐acceptor alternating vinyl bonds enables the synthesis of copolymers containing electron‐rich monomers that are too unreactive to readily homopolymerize and incorporate a functional handle in every MAnh repeat unit. The anhydride functionalities can readily be modified when in the presence of various nucleophiles to afford imides, amides, esters, or acids, where they influence polymer solubility and other properties. The strong alternating tendency and facile functionalization make MAnh an attractive electron‐deficient monomer in radical polymer chemistry (Figure 3).

### 2.2 Controlled Radical Polymerization (CRP)

#### 2.2.1 Atom Transfer Radical Polymerization (ATRP)

To date, MAnh has not been copolymerized via ATRP as a result of unfavourable interactions between the monomer and the required metal catalyst [71, 72]. This is primarily an issue for copper‐mediated ATRP, as the carboxylic acids of residual ring‐opened MAnh react with the Cu(II) species [73, 74]. In this event, the carboxylic acid groups displace the halogen atom (often bromine), resulting in the formation of a metal carboxylate complex and ultimately inhibiting polymerization [73, 74]. To circumvent this functional group sensitivity, researchers have historically modified the anhydride functionality, i.e., conversion of the anhydride moiety to an imide [72]. The modification results in a compromise, though, as the new monomer will have different reactivities during copolymerization and as it cannot be easily modified back to an anhydride post‐polymerization, it cannot be seen as a feasible protection strategy.

#### 2.2.2 Nitroxide Mediated Polymerization (NMP)

Although ATRP has not been successfully conducted with MAnh, there are instances where MAnh has been copolymerized via NMP. In all these instances, styrene was copolymerized with MAnh [71, 75, 76, 77, 78, 79, 80]. The utilization of NMP and an excess of styrene resulted in the generation of block copolymers, with the first block consisting of an alternating copolymer of poly(styrene‐*co*‐maleic anhydride), SMAnh, and the second block of polystyrene. All blocks possessed low dispersity and high chain‐end fidelity as confirmed by chain extension [71]. Work by Lessard et al. improved upon the initial work by Hawker and coworkers with their utilization of the versatile BlockBuilder initiator, whereby a block copolymer with low dispersity was generated [77].

#### 2.2.3 Reversible Addition Fragmentation Chain‐Transfer(RAFT)‐Mediated Polymerization

**FIGURE 4:**
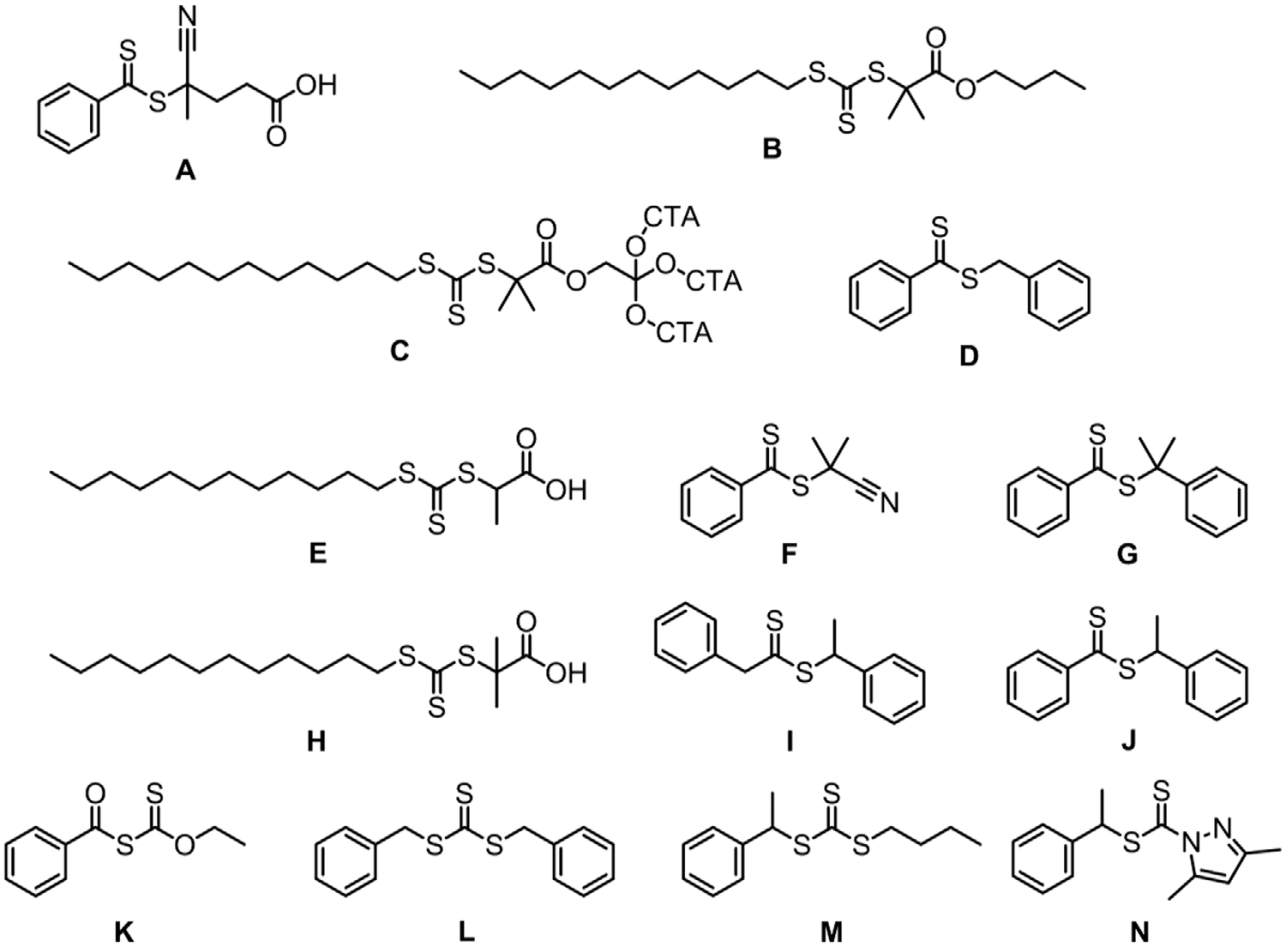
Chain transfer agents (CTAs) utilized in the copolymerization of MAnh with various donor comonomers.

RAFT‐mediated copolymerization is the most prevalent controlled polymerization technique utilized in conjunction with MAnh. In most scenarios, styrenic monomers have been copolymerized with MAnh, particularly via the utilization of dithiobenzoate (DTB) and trithiocarbonate (TTC) chain transfer agents (CTAs) [73, 81, 82, 83, 84, 85]. A recent study by Ball et al. highlights the first implementation of dithiocarbamate (DTC) CTAs for the controlled synthesis of SMAnh (Figure 4N) [82]. More recently, a study by Ball et al. describes the optimal CTA Z‐group class for SMAnh copolymerization (and bioderived alternatives) [86]. It was noted that all three above‐mentioned Z‐group classes are suitable for the SMAnh system, whereas xanthates show an inferior degree of control [87].

Due to the difference in electron density of MAnh and electron‐rich comonomers, the two comonomers (MAnh and donor monomer) behave differently in copolymerization reactions. In a study by van den Dungen et al., the initialization process of the copolymerization of styrene and MAnh with two DTBs, **F** and **G** (Figure 4), was investigated [83]. It was noted that the RAFT agent leaving group had specificity in its addition of either styrene or MAnh, where CTA **F** (Figure 4) preferentially underwent the addition of styrene compared to MAnh. Conversely, CTA **G** (Figure 4) preferentially added MAnh during the first monomer addition. In studies by Ball et al. and Smith et al., it was highlighted that the terminal monomer identity of a styrene‐MAnh copolymer impacts both the chain extension [82] and thiocarbonylthio group removal kinetics [88], respectively. It is therefore of paramount importance to characterize and note the identity of the terminal monomer present on MAnh‐containing copolymers, as this has been shown to have implications on polymerization (initialization, chain extension, and thiocarbonylthio group thermal stability) and thiocarbonylthio reaction kinetics. Furthermore, a study by Charleux and coworkers highlighted that the comonomer ratio can impact the RAFT‐mediated copolymerization kinetics and molecular weight distribution (MWD) of the resulting MAnh‐containing copolymer [89]. More specifically, faster copolymerizations with broader MWDs were observed when the fraction of MAnh in the monomer feed was higher [89]. This was hypothesized to be a result of a decrease in the chain transfer constant of CTA **D** (Figure 4), and an increase in the average rate coefficient of propagation with a larger fraction of MAnh in the feed [89].

There are a few instances where other comonomers have been utilized in conjunction with MAnh, i.e., acrylonitrile (Figure 4A), where narrow MWDs (*Ð* <1.3) and conversions (α) of around 0.55 were acquired with CTA **A** (Figure 4) [90]. Notably, β‐pinene has been successfully copolymerized with MAnh (molar ratio of 3:7) with α = 0.2 – 0.3 (*Ð* <1.3) (Figure 4I,J) [91]. Livingness (narrow MWD and chain end retention) of the RAFT‐mediated copolymerization was confirmed by chain extension with styrene to afford poly(β‐pinene‐*alt*‐maleic anhydride)‐*b*‐polystyrene. Recently, photo‐induced RAFT (PI‐RAFT)‐mediated polymerization was conducted on MAnh and 1, 3‐pentadiene (cis, trans, and both) (Figure 4K) [92]. During this photo‐mediated polymerization, α = 1.0 was achieved in 6 h (*Ð* ∼1.39) [92]. MAnh has also been utilized in terpolymerizations, as highlighted in studies reported by Hu and Zhang [93]. Styrene, MAnh, and *N*‐vinyl‐pyrrolidone were copolymerized with CTA **L** (Figure 4) [93]. The monomers used in this work led to α in excess of 0.9 (*Ð* = 1.20 – 1.47). When MAnh is copolymerized with other comonomers (instead of styrene), TTCs and DTBs are the dominant CTAs utilized in conjunction with either benzylic, secondary, or tertiary R groups. It may be of further interest to investigate the possibility of utilizing DTCs with other comonomer pairs containing MAnh, as this has shown success when MAnh is copolymerized with styrene.

Work by Sumerlin and coworkers highlights the ease of modification of SMAnh, where a one‐pot synthesis of poly(styrene‐*co*‐maleic anhydride)‐*b*‐polystyrene was carried out with the aid of CTA **H** (Figure 4) [94]. Thereafter, post‐polymerization modification was conducted on the MAnh repeat units with furfurylamine to introduce suitable functionalities for crosslinking with bismaleimide‐containing molecules [94]. These responsive polymers could be applied in the lubricant additive industry, as well as in the coatings field [94]. This one‐pot block copolymer formation was also achieved with CTA **I** (Figure 4) by Yao et al. [95]. Another example of ease of crosslinking is highlighted in the work by Benvenuta‐Tapia et al. [96], whereby an elastomeric thermoplastic multiblock copolymer was developed for use as an asphalt modifier. The multiblock copolymer consisted of a middle poly(butyl acrylate) block, followed by polystyrene and poly(styrene‐*co*‐maleic anhydride) to afford SMA‐S‐BA‐S‐SMA block copolymers (CTA **L**, Figure 4) [96]. The incorporation of MAnh units into the polymer afforded chemical interactions with the asphaltenes in the asphalt, resulting in a polymer with enhanced compatibility and performance compared to non‐functionalized copolymers or unmodified asphalt. Work by Perrier and coworkers emphasizes that controlling the percentage of MAnh incorporated into the polymer backbone can be exploited to control the crosslinking ability/density of copolymers [97]. The more MAnh units within the polymer backbone, the greater the crosslinking density between polymers. This work was completed by the sequential one‐pot polymerization of styrene blocks and MAnh single monomer insertions (CTA **B**, Figure 4) [97]. It is therefore possible to modulate the physical properties of MAnh‐containing copolymers in a facile manner by modifying the MAnh repeat unit with a nucleophilic moiety containing variable length, order/disorder, or hydrophilicity/hydrophobicity. An example of such an instance is found in a study by Perrier and coworkers, where the authors synthesized a library of SMAnh copolymers (with varying comonomer feed) with different architectures (linear and 4‐arm star) with CTAs **B** and **C** (Figure 4). The MAnh repeat units were thereafter ring‐opened with C_22_ alcohols to afford copolymers with different grafting densities based on the MAnh content in the copolymer [98]. The side chain density and distribution were noted to impact the polymer crystallization temperatures.

MAnh presents several challenges when utilized in conjunction with different CRP techniques. MAnh currently remains incompatible with ATRP due to its sensitivity toward copper catalysts, while NMP provides limited use, but reliable access to SMAnh block copolymers. Contrarily, RAFT has been shown to be the most robust approach to afford control over MWD, architecture, and functional density. Most prominently, the choice of CTA and the terminal monomer identity impact polymerization kinetics, livingness, and post‐polymerization reactivity of the copolymer. MAnh, therefore, can afford the generation of precise macromolecules with functionalization potential.

## 3 Polymer Modification or Monomer Synthesis

**FIGURE 5:**
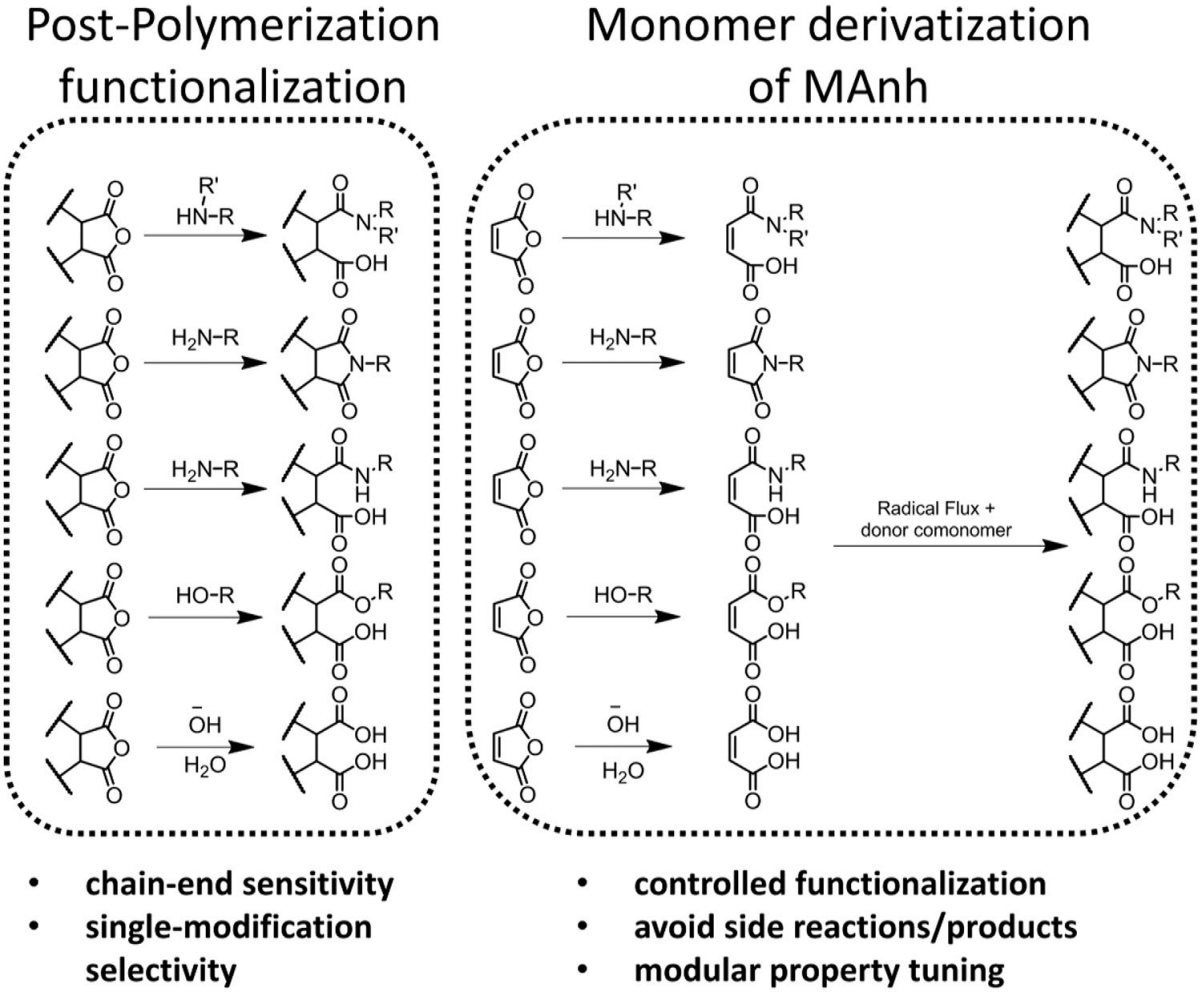
A comparison of pre‐ and post‐modification of anhydride functionalities in MAnh.

MAnh repeat units are easily modified via post‐polymerization techniques for various applications such as crosslinking [65]. For some applications, however, such post‐polymerization modifications may not be ideal due to 1) chemical sensitivity, i.e., the modification of MAnh with a primary amine may result in aminolysis of the RAFT chain‐end functionality, which is detrimental for chain extension reactions [99], or 2) selectivity, i.e., the incorporation of a single modification per copolymer chain (Figure 5). The following section focuses on several monomeric MAnh derivatives and their implementation to date. It is worth noting that each derivative will result in a different polymer with different properties and thus should be carefully selected based on the properties imparted compared to the original unmodified monomer (MAnh).

## 4 Maleimide (MI)

**FIGURE 6:**
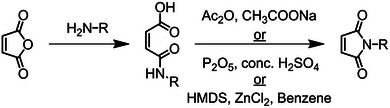
Methods for the synthesis of maleimides from MAnh. Different approaches can be conducted based on the pendant group to be attached [100, 101, 102, 103].

Maleimides (MIs) are the most studied derivative of MAnh. They are synthesized via the addition of a primary amine to the anhydride moiety, followed by ring closure to form the respective MI (Figure 6) [100, 101, 102, 103]. A second method involves first protecting the vinyl bond via a Diels‐Alder reaction of maleic anhydride with furan, and thereafter the functionalization of the monomer and subsequent ring closure. Following the functionalization of the monomer, the furan protecting group is removed [104]. These facile approaches to monomer synthesis afford the generation of a broad variety of *N*‐substituted MI derivatives. Like MAnh, MI is an electron‐deficient monomer that tends to alternating comonomer incorporation when in the presence of electron‐donor monomers. However, unlike MAnh, MIs are capable of undergoing homopolymerization [105, 106, 107, 108]. Furthermore, in contrast to MAnh, which is sensitive to moisture, metal cations, and nucleophiles, MIs show minimal sensitivity and higher hydrolytic stability, but often invoke greater hydrophobicity within the polymer chain, dependent on the substituent on the nitrogen atom of the MI, designated as R (Figure 6) [109].

### 4.1 Conventional Radical Polymerization

**FIGURE 7:**
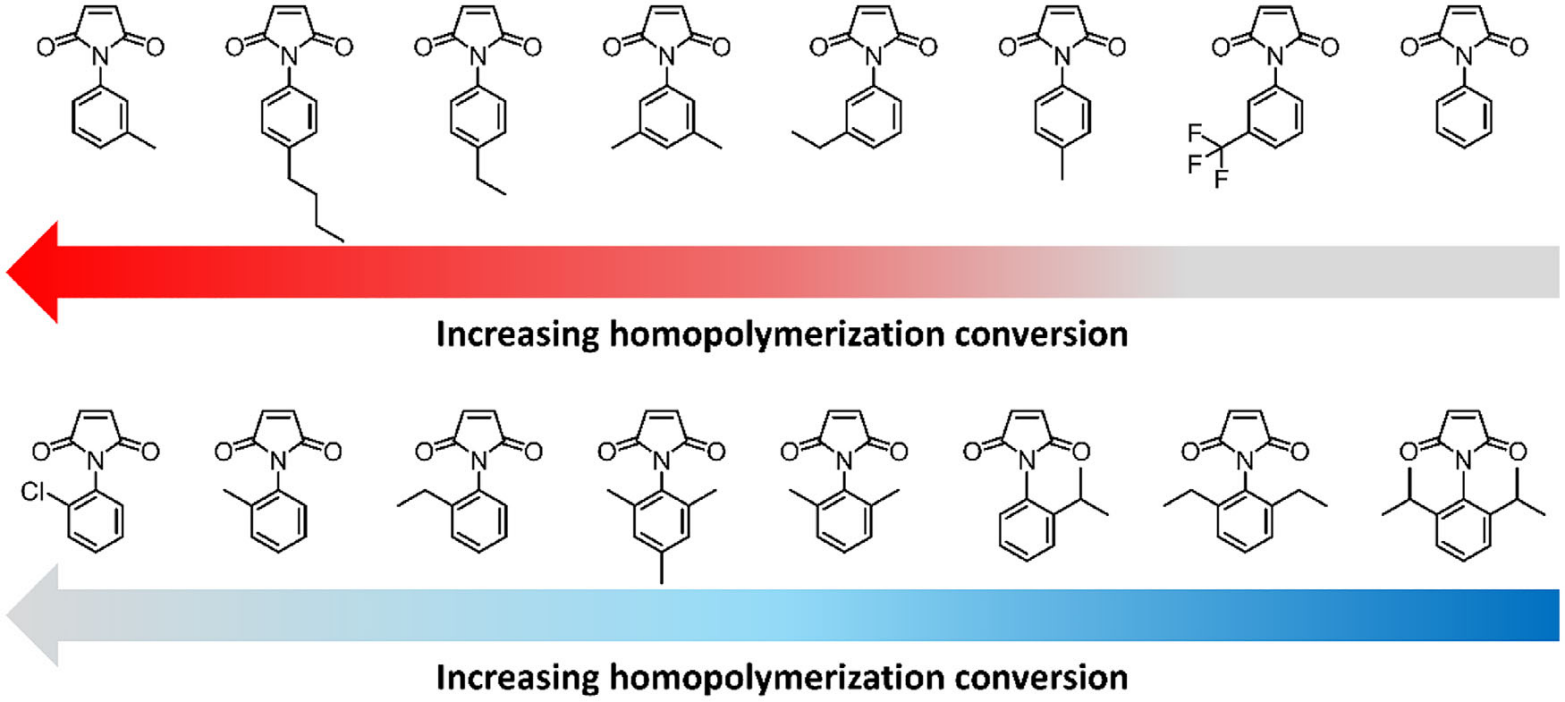
N‐(substituted phenyl) maleimide library synthesized by Matsumoto et al. [108] From top left to bottom right, the conversion during homopolymerization of the maleimide derivatives decreases.

Compared to MAnh, MIs are capable of homopolymerization under relatively mild conditions (∼60°C, AIBN) [105, 106, 107, 108, 110]; however, they will readily undergo copolymerization when in the presence of a donor (electron‐rich) monomer. As with MAnh, MI can undergo photoinitiation (in conjunction with a donor comonomer) as a result of the electron‐deficient alkene, which results in auto‐initiation when the comonomer pairs are irradiated [111, 112]. There have been several studies by Matsumoto and co‐workers investigating the impact of steric effects on the homopolymerization of *N*‐phenyl maleimide derivatives [107, 108, 113]. It was found that an increase in the steric interactions at the *ortho* position retarded the rate of homopolymerization, while the introduction of alkyl groups in the *meta* and *para* positions increased the rate of homopolymerization (inductive effect) (Figure 7) [108].

**FIGURE 8:**
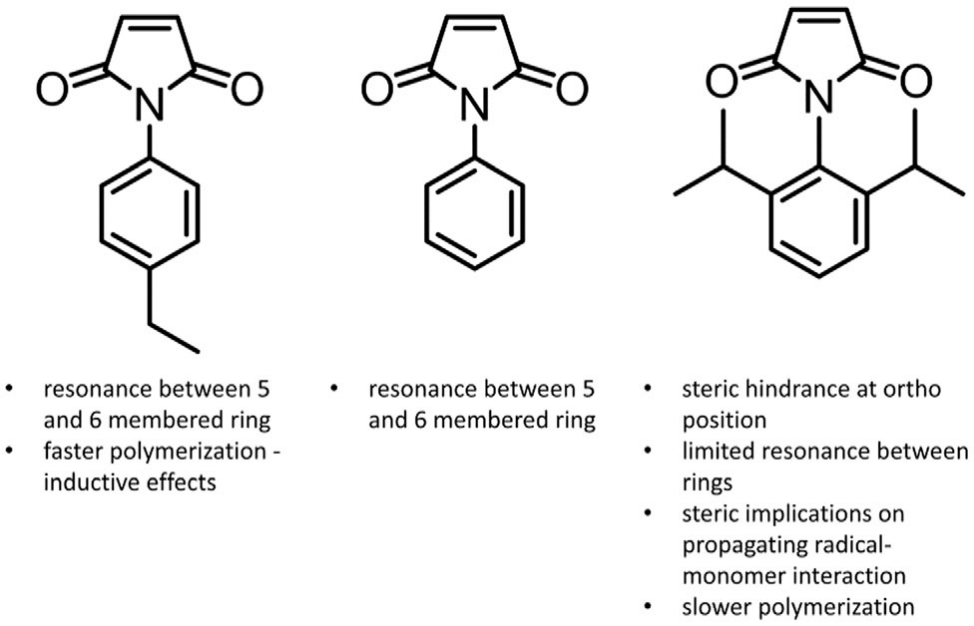
Impact of substitution patterns on *N*‐phenylmaleimides.

The steric effects of the *ortho*‐alkyl substituents result in the displacement of the aromatic ring so that it is perpendicular to the 5‐membered ring of the MI. This was hypothesized to limit the rate of polymerization and potentially prohibit resonance between the 6‐membered and 5‐membered rings, thereby lowering monomer reactivity. Furthermore, steric hindrance of the alkyl groups in the *ortho* position may block the propagating radical from reacting with monomers in solution (Figure 8). [108] Further investigations highlight that the introduction of electron‐withdrawing groups on the aromatic ring decreases the rate of polymerization. A subset of this monomer library was copolymerized with styrene (affording alternating copolymers) and/or methyl methacrylate [108]. It was noted that MIs with substituents in the *ortho* position partook in copolymerizations at significantly lower rates of monomer addition. Lastly, the steric bulk of the MI substituent can impact the sequence control of specific comonomer pairs as indicated by Matsumoto and Terada, Masuda et al., and Hisano et al. [114, 115, 116]. Due to MIs tolerance for functional groups and polymerization conditions, it has been copolymerized with a wide variety of comonomers, such as styrenic [117, 118, 119, 120, 121, 122, 123], methyl methacrylate [124], methacrylate (hydroxypropyl and azobenzene) [125, 126], dienes [43, 127, 128], vinyl ether [129, 130, 131, 132], *N*‐vinylpyrrolidone [133, 134], olefin [114, 115, 116, 135, 136, 137], selenophene [138], and vinyl ethylene carbonate [139, 140]. Interestingly, MI is one of the few monomer classes in this review that has been utilized as a comonomer in radical ring‐opening copolymerization, primarily in conjunction with cyclic ketene acetals [141, 142, 143].

### 4.2 CRP

#### 4.2.1 ATRP

In contrast to MAnh, MIs are regularly utilized in ATRP‐mediated copolymerizations because of the imide functionality, which is not a carboxylic acid precursor. The majority of these copolymerizations make use of styrene as the comonomer [72, 144, 145]. This is a logical choice due to the electron‐rich/electron‐poor character of the comonomer pair. It would be interesting to investigate other donor comonomers, e.g., vinyl acetate and *N*‐vinylpyrrolidone, to increase the diversity of accessible copolymers in terms of hydrophilicity/hydrophobicity. There is a single instance in literature where organocatalyzed ATRP was utilized to homopolymerize and copolymerize MI derivatives [146]. It would be interesting to apply this system to the copolymerization of MAnh with other donor monomers, as there is no instance in literature where ATRP has been successfully conducted on a donor‐MAnh copolymerization system.

#### 4.2.2 NMP

Following a similar trend as ATRP for MIs, most work reported for NMP has been explored in conjunction with the copolymerization of styrene [147, 148, 149]. Although there has been work conducted in this field, it is rather minimal in comparison to the number of studies reported for other CRP methods of this monomer class.

#### 4.2.3 RAFT

RAFT is the primary mode of controlled radical (co)polymerization for MIs, where a significant proportion of this research focuses on radical ring‐opening copolymerization (with MI as an electron‐poor comonomer to encourage the RROP) [141, 142, 150], copolymerization with naturally derived comonomers [115, 137, 151, 152], and research into monomer sequence regulation [153, 154, 155, 156].

**FIGURE 9:**
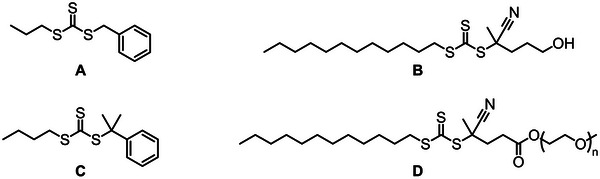
Some CTAs utilized in the RAFT‐mediated synthesis of polymers containing MIs.

It is interesting to note that these comonomers primarily utilize DTBs (Figure 4G,J) [153, 155, 156, 157], and TTCs (Figures 4H and 9A–D) [115, 137, 141, 142, 150, 151, 152, 154]. For future research, it would be worth investigating the utilization of DTCs due to their recently published utility in the copolymerization of MAnh [86].

**FIGURE 10:**
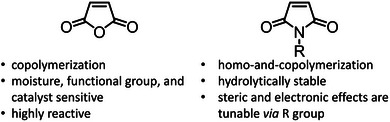
Comparison of monomer properties of MAnh and MI.

MIs provide greater hydrolytic stability than MAnh, with the capacity to homopolymerize while maintaining an alternating tendency of incorporation when copolymerized with electron‐rich monomers. Their reduced sensitivity toward nucleophiles and metal ions (compared to MAnh) increases their compatibility across various CRP techniques, which are otherwise inaccessible to MAnh. Importantly, the substituent on the nitrogen atom has been shown to strongly dictate monomer reactivity, solubility, and polymer properties. MIs, therefore, are positioned as MAnh alternatives with increased stability, functional tolerance, and versatility (Figure 10).

## 5 Maleic Acid (MA) and Fumaric Acid (FA)

Compared to MAnh, its hydrolysed derivatives, maleic (Z isomer, MA) and fumaric (E isomer, FA) acid, are seldom utilized in the literature. This is intriguing when considering the extensive utilization of acrylic acid in aqueous polymerizations to afford hydrophilic polymers and noting that the di‐acid functionality often exploited in styrene‐MA copolymer applications is often afforded via the post‐polymerization hydrolysis of MAnh repeat units; therefore, it is ideal to directly utilize the di‐acid form in copolymerizations to avoid unnecessary synthetic steps. Both di‐acid monomers are derived from the hydrolysis of MAnh, although they can both be readily acquired through commercial suppliers. While maleic acid is produced purely via petrochemical means, fumaric acid can be produced on a large scale via fermentation‐based methods [158]. Understandably, the isomers behave differently during copolymerizations based on the varying orientations of the carboxylic acid substituents (E vs Z). FA, the more stable E isomer, will incorporate at a slower rate than the MA derivative [159, 160]. The differing reactivities could afford copolymers that are dissimilar in monomer sequence distribution and composition, and are worth investigating in the future.

### 5.1 Conventional Radical Polymerization

There is no evidence in the literature that fumaric acid or maleic acid are found to homopolymerize. Instead, the monomers are utilized in copolymerizations as shown in a collection of comparative studies by Zeliazkow, where both FA and MA were copolymerized with styrene [159, 160, 161]. In addition to styrene, there have been several other monomers copolymerized with both FA (*N*, *N*‐diallyl‐*N*, *N*‐dimethylammonium chloride [162], and 2, 2‐diallyl‐1, 1, 3, 3‐tetraethylguanidinium chloride [163]) and MA (styrenics [164], acrylamides [165, 166], and *N*, *N*‐diallyl‐*N*, *N*‐dimethylammonium chloride [162]). Although little research has been conducted on these monomers, there has been a recent increase in the utilisation of maleic acid for its hydrophilic properties to make superabsorbent polymers [166], iron sensing and extraction [165], and the synthesis of 2D polymers [164].

### 5.2 CRP

A recent study was conducted on the RAFT‐mediated synthesis of poly(styrene‐*co*‐maleic acid) by Smith et al. [15]. The TTC (Figure 4M) was found to best mediate the copolymerization, affording material with narrow MWD (*Ð* = 1.27) and α = 0.30 compared to DTB (*Ð* = 1.34 and α = 0.21, Figure 4F) and DTC (*Ð* = 1.64 and α = 0.29, Figure 4N) as the CTA for this copolymerization. Furthermore, it was highlighted that the copolymerization kinetics of styrene and MA were significantly slower than those of styrene and MAnh. This was hypothesized to be a result of the differences in the radical reactivity of MA vs. MAnh during the propagation of the polymer chain end. The differences in the monomer electronics were reflected in the styrene‐MA copolymerization, obeying the terminal monomer model (no MA homopropagation) compared to the styrene‐MAnh copolymerization, which is historically known to obey the restricted penultimate model [12]. The lower tendency toward alternating copolymerization in the styrene‐MA case makes it possible to synthesize non‐alternating SMA with a narrow MWD, which has until now been proven impossible through the styrene‐MAnh copolymerization. Although MA is a good substitute for MAnh (affording copolymer with low molecular weight), it copolymerizes significantly slower than MAnh, which results in lower monomer conversion in 30 h. To afford this monomer as a replacement for MAnh in the synthesis of hydrophilic copolymers on a large scale, a copolymerization method would need to be developed to afford higher monomer conversions.

Based on recent discoveries in the copolymerization of MA, it would be interesting to expand the scope of this work and include FA copolymerizations. Studies on the relationship between monomer sequence distribution in the copolymer and monomer stereochemistry will contribute to the overall understanding of SMA and its derivatives. Furthermore, it would be of interest to investigate the monomer reactivity as a function of protonation state, as was previously investigated for acrylic acid [167].

## 6 Dialkyl Maleates (DAMs) and Dialkyl Fumarates (DAFs)

**FIGURE 11:**
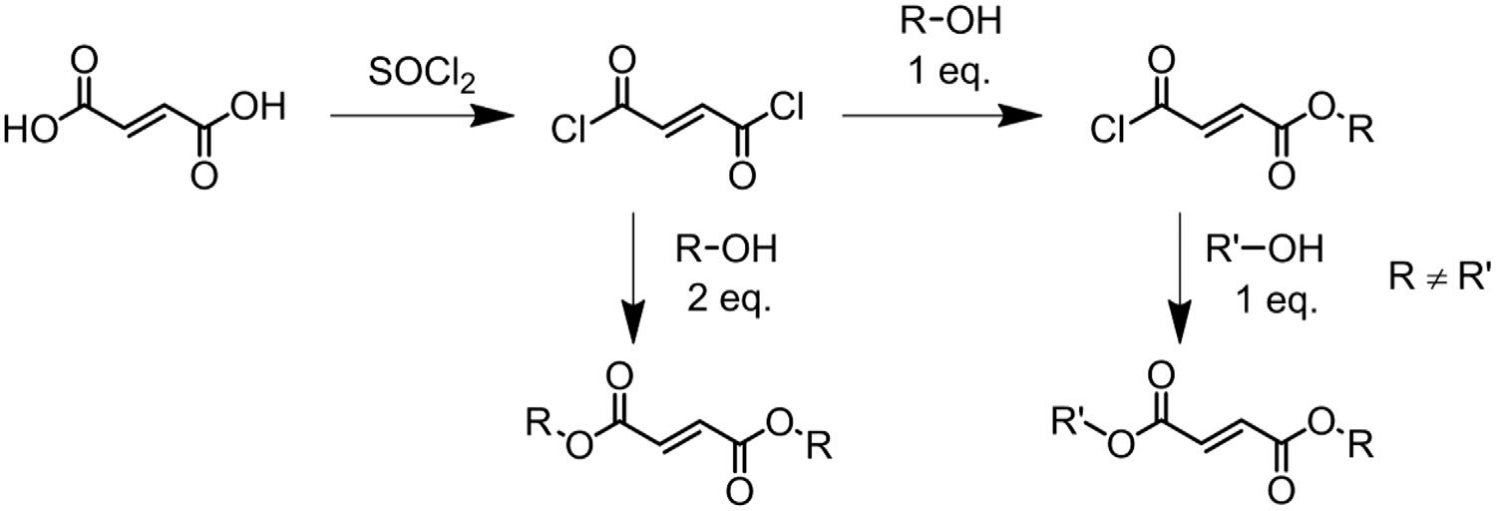
Synthetic route for the synthesis of fumarate derivatives from fumaric acid.

Dialkyl maleates (DAMs) and dialkyl fumarates (DAFs) are the most studied ring‐opened derivatives of MAnh. DAFs are easily synthesized via the addition of an alcohol to fumaryl chloride to afford either the symmetrically or asymmetrically substituted DAF (Figure 11) or via Fischer esterification of fumaric acid [168, 169]. Contrarily, the DAMs need to be synthesized with caution as the monomer may isomerize and, therefore, it is recommended to conduct the synthesis via DCC/DMAP coupling.

### 6.1 Conventional Radical Polymerization

**FIGURE 12:**
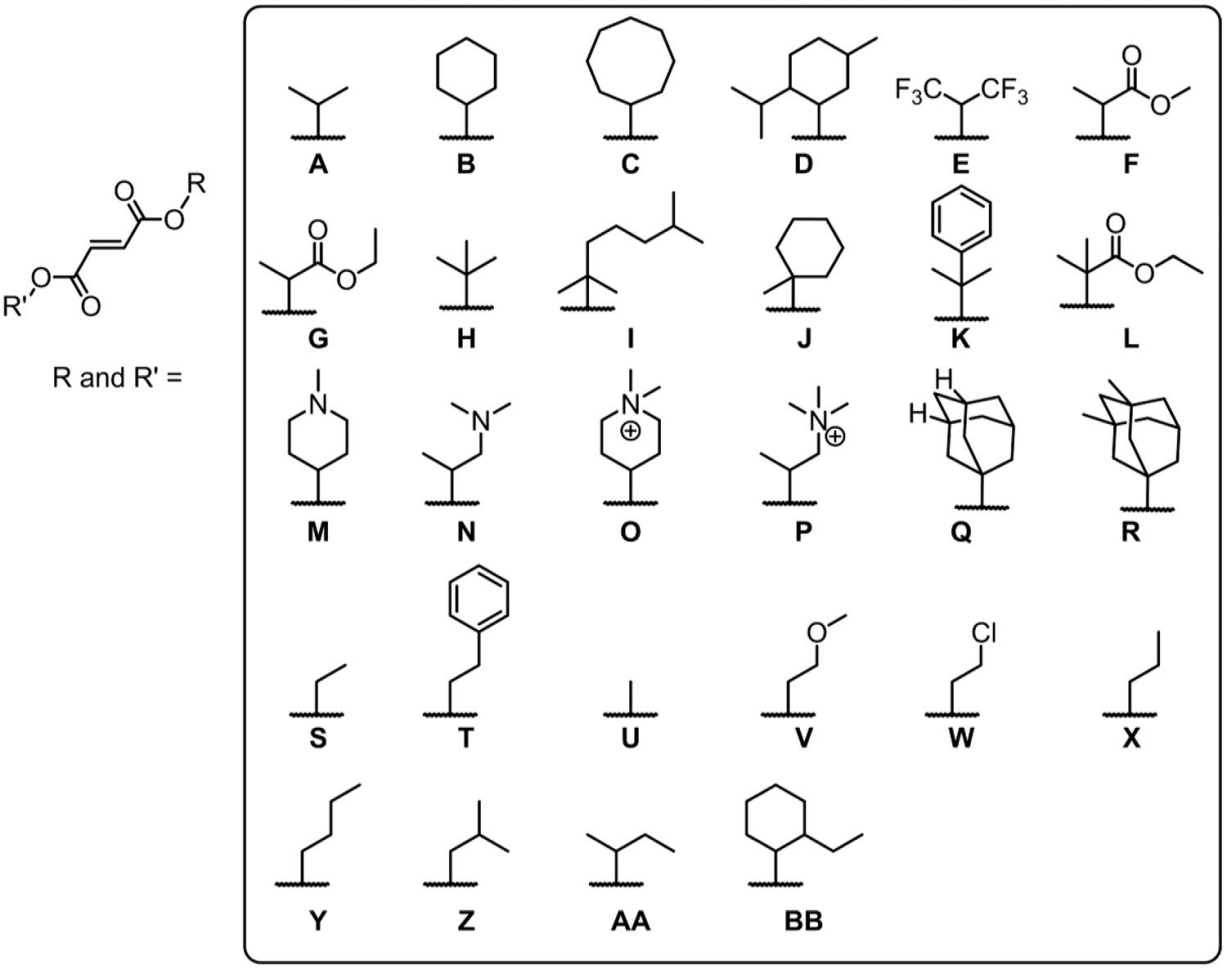
Some of the dialkyl‐fumarate (DAF) derivatives that have been synthesized and homopolymerized.

DAFs are easier to synthesize than DAMs and are therefore more extensively studied than DAMs in the literature. It is reported that DAFs with substituents of various lengths can readily homopolymerize (Figure 12) [170, 171, 172, 173, 174, 175, 176, 177, 178, 179, 180, 181]. Several studies have shown that the incorporation of larger substituents is beneficial for the homopolymerization of fumarates, whereby larger substituents increase k_p_ (**H**:0.61> **B**:0.60> **A**:0.46> **S**:0.21> **U**: 0.058, (L·(mol·s)^−1^), Figure 12, extracted from ESR data). Although k_p_ increases depending on the bulk of the substituent, the k_p_ is still significantly lower than that of methyl methacrylate (DAF k_p_ is 10^3^ times smaller, based on ESR data) [181]. The increase in substituent steric bulk was also noted to retard chain termination as a result of the increased steric repulsion, evident via ESR data (i.e., **U**: k_t_ = 430 L·(mol·s)^−1^, **H**: k_t_ = 30 L·(mol·s)^−1^, Figure 12) [174, 175, 182]. Therefore, although the k_p_ for DAFs is lower (by approximately three orders of magnitude) than more commonly utilized monomers like methyl methacrylate, the ratio of k_p_ to k_t_ is such that high degrees of polymerization can be obtained (confirmed via SEC data) [181]. Furthermore, in work by Pantoustier and coworkers [172], it was highlighted that the homopolymerization of fumarates is functional group sensitive, with carbon based substituents having limited impact on the polymerization conversion (i.e. **E**—**G** and **K**—**P** = α <0.1, and **A**—**D** and **H**—**J** = α > 0.3, Figure 12) [172].

Beyond substituent effects, the choice of radical initiator can significantly impact the homopolymerization of DAFs. Yoshioka and Otsu et al. [183, 184, 185], report that although both radical sources have similar decomposition rates (k_d_ = 8.4×10^−6^ s^−1^ for MAIB at 60°C and k_d_ = 9.8×10^−6^ s^−1^ for AIBN at 60°C), it was noted that AIBN had a lower initiation reactivity (participated in primary radical termination and inactivation events) compared to MAIB, which only initiated polymerization. The disparity in radical activities and reaction events results in the different polymerization kinetics observed with different radical sources. This effect was not observed for standard monosubstituted vinyl monomers. It is therefore recommended to make use of MAIB and not AIBN for the initiation of DAFs. In addition to their homopolymerization capabilities, DAFs can copolymerize with several monomers such as carbazoles [186], vinyl ethers [187, 188], acrylamide [189], acrylates [190], and styrenics [177, 191, 192].

Unlike DAFs, maleates (DAMs) do not homopolymerize unless in the presence of an isomerization catalyst (amine), which effectively results in the homopolymerization of DAFs [193, 194, 195]. Although the monomer class has limited use in homopolymerizations, DAMs have been copolymerized with several comonomers, such as vinyl ethers [51, 187, 196, 197, 198], styrene [192, 199, 200, 201], methyl methacrylate [202], vinyl acetate [203, 204], and vinyl carbazole [205]. It is believed that these monomers follow similar trends to those of the DAFs, with larger substituents resulting in higher monomer conversion as a result of reduced termination rates, although further studies are required.

Substituent size plays a crucial role in controlling propagation and termination kinetics for both dialkyl maleates and fumarates. Ultimately, the steric hindrance of the pendant groups can be utilized as a design feature to tune monomer reactivity in polymerizations. Although DAFs exhibit slower propagation kinetics compared to other monomer classes, the favourable k_p_/k_t_ ratios still enable the synthesis of moderate molecular weight polymers, which would be ideal for applications requiring (co)polymers with rigid backbones.

### 6.2 CRP

#### 6.2.1 ATRP

No research has currently been published on the ATRP of maleates, neither in homopolymerization nor in copolymerization. Contrarily, fumarates have been investigated by Matsumoto et al., where it was noted that the ATRP methods were not suitable for the homopolymerization of diisopropyl‐fumarate, with low polymer yields and with molecular weights that exceeded the theoretically predicted values [206]. This was hypothesized to be a result of bimolecular termination events and the concomitant accumulation of Cu(II), which would ultimately suppress the polymerization.

#### 6.2.2 NMP

While no recent studies have been conducted on the NMP of maleates, some research has been reported previously by Matsumoto et al. [206]. In a similar narrative to ATRP for this monomer class, NMP was inefficient in controlling the homopolymerization of the fumarate derivative. Irrespective of various initiators, the molecular weights of the isolated polymer were similar to those of systems without nitroxide species present in the polymerization. This further confirms the lack of control observed in the polymerization, which is hypothesized to be a result of insufficient initiation and the presence of significant irreversible termination events.

#### 6.2.3 RAFT

**FIGURE 13:**
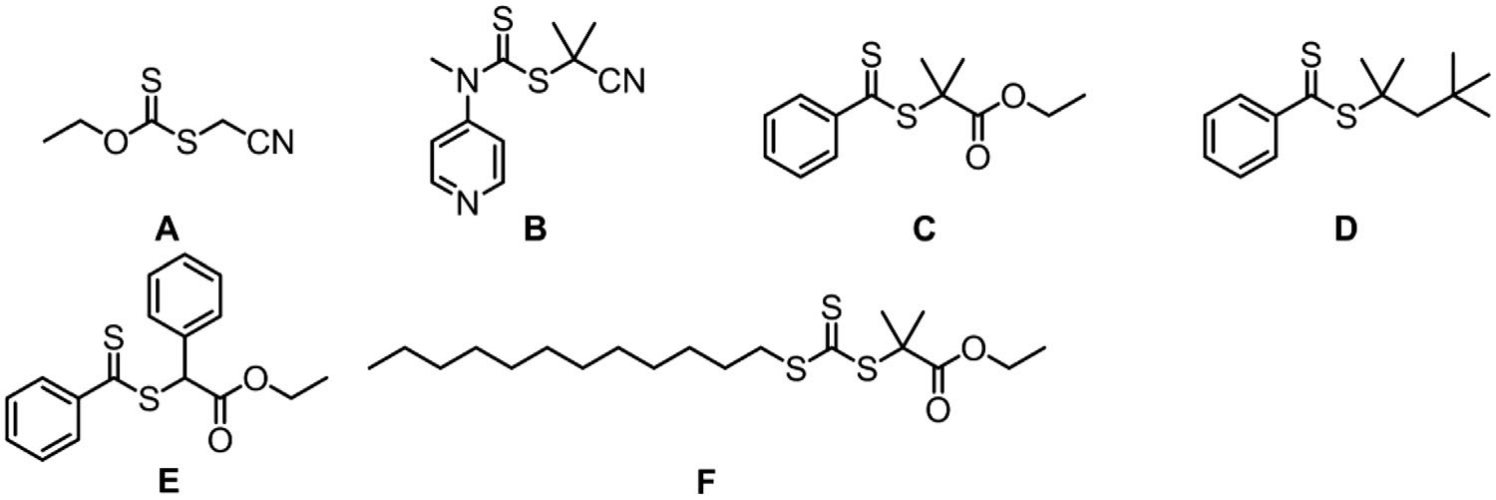
CTAs utilized to synthesize (co)polymers containing maleates and fumarates.

Maleates have been copolymerized with vinyl ethers, using xanthates and DTCs (Figure 13A,B) [207, 208]. It is noted that the MWD acquired from these copolymerizations was broader than expected for RAFT processes (*Ð* > 1.4), and although CTA retention is confirmed via chain extension, it is accompanied by a further broadening of the MWD. This may be because of partial bimolecular termination occurring at high α (0.5) and is further evident when higher molecular weights (M_n_) are targeted. Therefore, the deviations from ideal behaviour are most likely caused by termination events [206]. Compared to other CRP techniques, RAFT has been the most successfully applied technique to synthesise (co)polymers of fumarates [206]. The majority of RAFT processes make use of DTBs (Figures 4G,J and 13C–E) [206, 209, 210], and TTCs (Figure 13F) [209]. In a study looking at different DTBs [210], the R group identity had a significant impact on the fragmentation and reinitiation rates, where aryl R groups led to poor control over the polymerization, i.e., CTAs **E** (Figure 13) and G (Figure 4), while polymerizations mediated by CTAs **C** and **D** (Figure 13) showed good control. Overall, CTA **C** (Figure 13) proved the best DTB to mediate the polymerization. In a later study [209], CTA **C** was compared to TTC **F (**Figure 13), where it was noted that TTC **F** outperforms DTB **C** in terms of faster kinetics and narrower MWD [209]. Therefore, the most optimal RAFT CTA class to date for fumarates is TTCs.

#### 6.2.4 Other CRP Techniques

It is worth acknowledging that other CRP techniques have been utilised on DAFs and DAMs, i.e., organotellurium‐mediated living‐radical polymerization (TERP) [206], iodine transfer radical polymerization (ITP) [211, 212, 213], and reversible chain transfer‐catalyzed polymerization (RTCP) [206]. TERP and RTCP were successfully conducted to generate moderately narrow MWD (1.2–1.4) poly(diisopropyl fumarate) [206]. The current methodology is limited to low molecular weights (∼5000 g/mol), affording high monomer conversion, although attaining lower M_n_ values compared to theoretical M_n_. Higher targeted M_n_ values afforded broad MWD and are therefore an avenue for future investigation [206]. Although with its limitations, TERP and RTCP seem significantly more promising as alternative CRP techniques than ITP. Current ITP studies are focused on the copolymerization of vinyl acetate with dibutyl maleate and were unsuccessful in attaining narrow MWD material (*Ð* > 1.4) [211, 212, 213]. Instead, we recommend that the community utilize xanthates to copolymerize vinyl acetate with dibutyl maleate to possibly afford a narrow MWD polymer.

## 7 Dialkyl Maleamide (DAMA), Dialkyl Maleamide‐Ester (DAMAE), Dialkyl Fumaramide (DAFA), and Dialkyl Fumaramide‐Ester (DAFAE)

**FIGURE 14:**
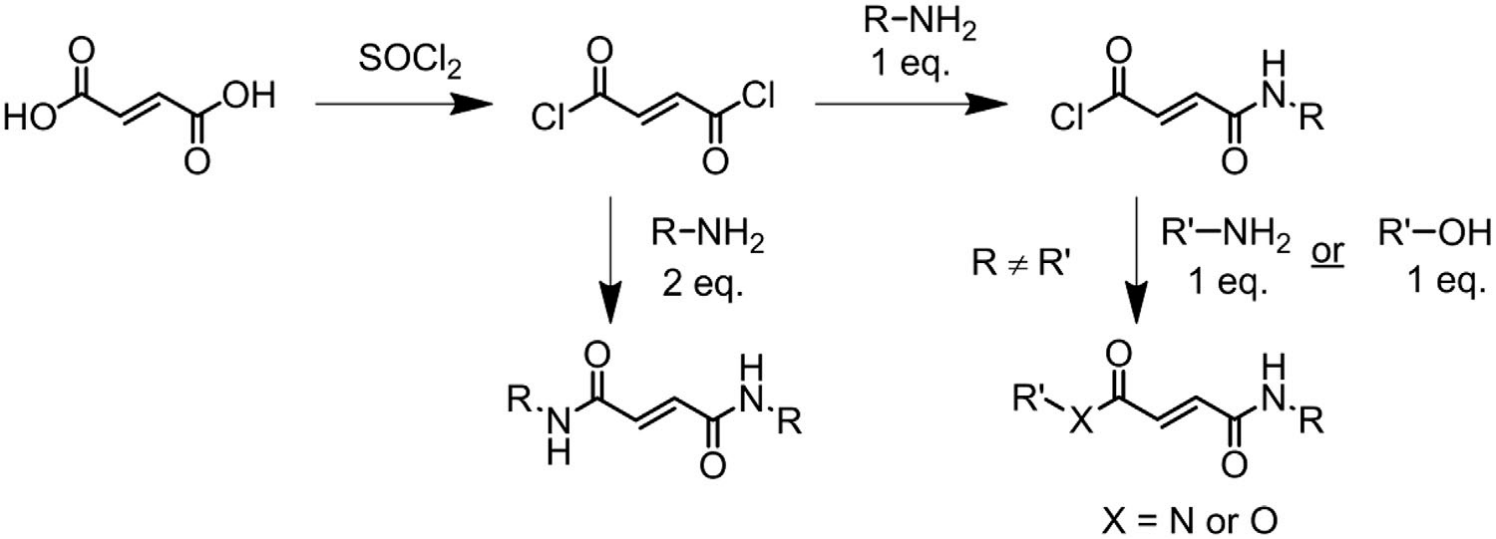
Recommended synthetic pathway to afford a desired fumaramide from fumaric acid.

As with maleates and fumarates, these monomers could generate polymers with significant backbone rigidity because of the di‐amide (or amide‐ester) repeat unit. Furthermore, the amide functionality could further impart directional stability via hydrogen bonding. A possible synthetic strategy to afford the monomers is stipulated in Figure 14. Fumaramides are simpler to synthesize as the Z isomer has larger stability than the E isomer (maleamide). Fumaryl chloride can be synthesized or bought and, thereafter, functionalized with either 1 or 2 equivalents of the desired amine to afford either a di‐hetero or homo‐substituted monomer. Subsequently, the desired product can be isolated via vacuum distillation, sublimation, or column chromatography [214, 215]. The amide‐ester derivative can be afforded via the addition of the relevant alcohol in the second addition as a replacement for the amine. If the R groups added to fumaric acid are too large (Figure 14), it may be necessary to investigate other coupling methods such as NHS/EDC, DCC/DMAP [216], or ethyl chloroformate coupling systems [217].

**FIGURE 15:**
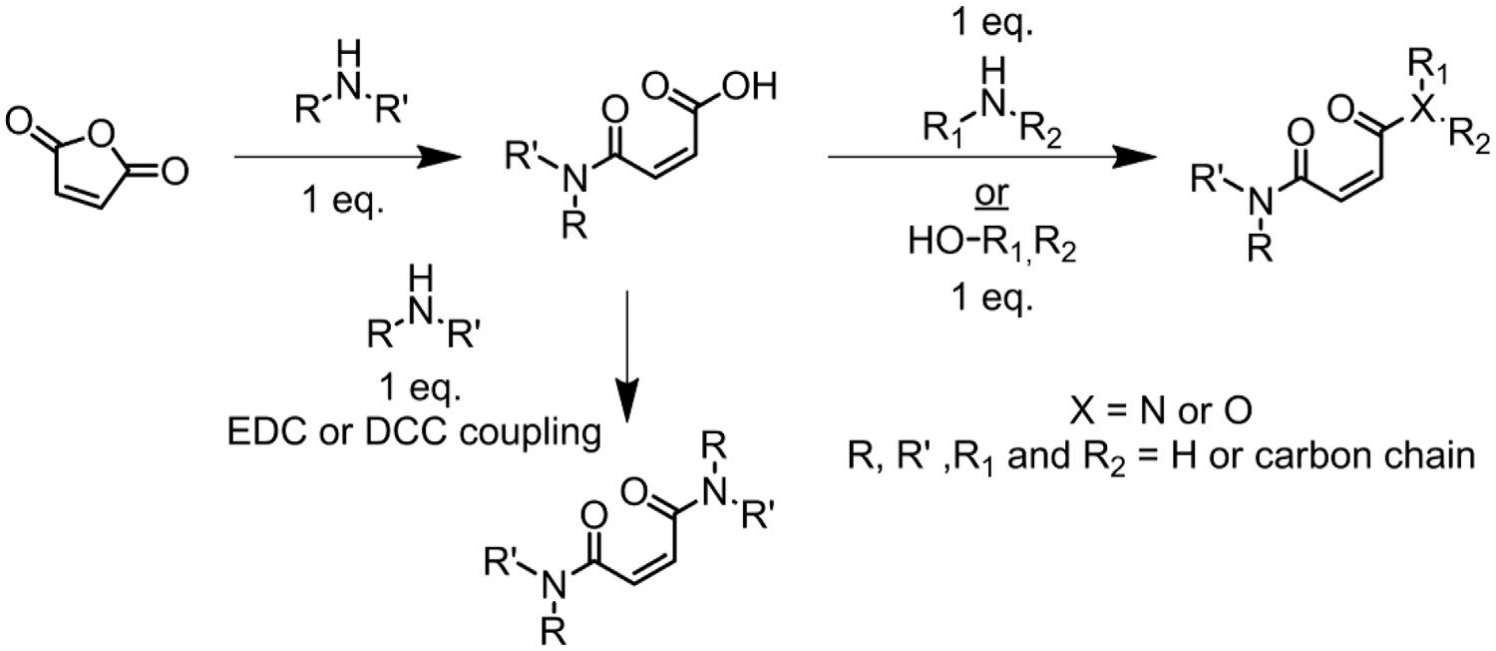
Recommended synthetic pathway to afford a desired maleamide from maleic anhydride.

In comparison, maleamide derivatives are more delicate to synthesize due to instances of isomerization to the more stable E isomer. A methodology for monomer synthesis can be found in the work by Majce et al. [218]. First, nucleophilic attack of MAnh with an amine occurs, followed by EDC coupling to the free carboxylic acid with an amine (Figure 15) [217, 218].

### 7.1 Conventional Radical Polymerization

**FIGURE 16:**
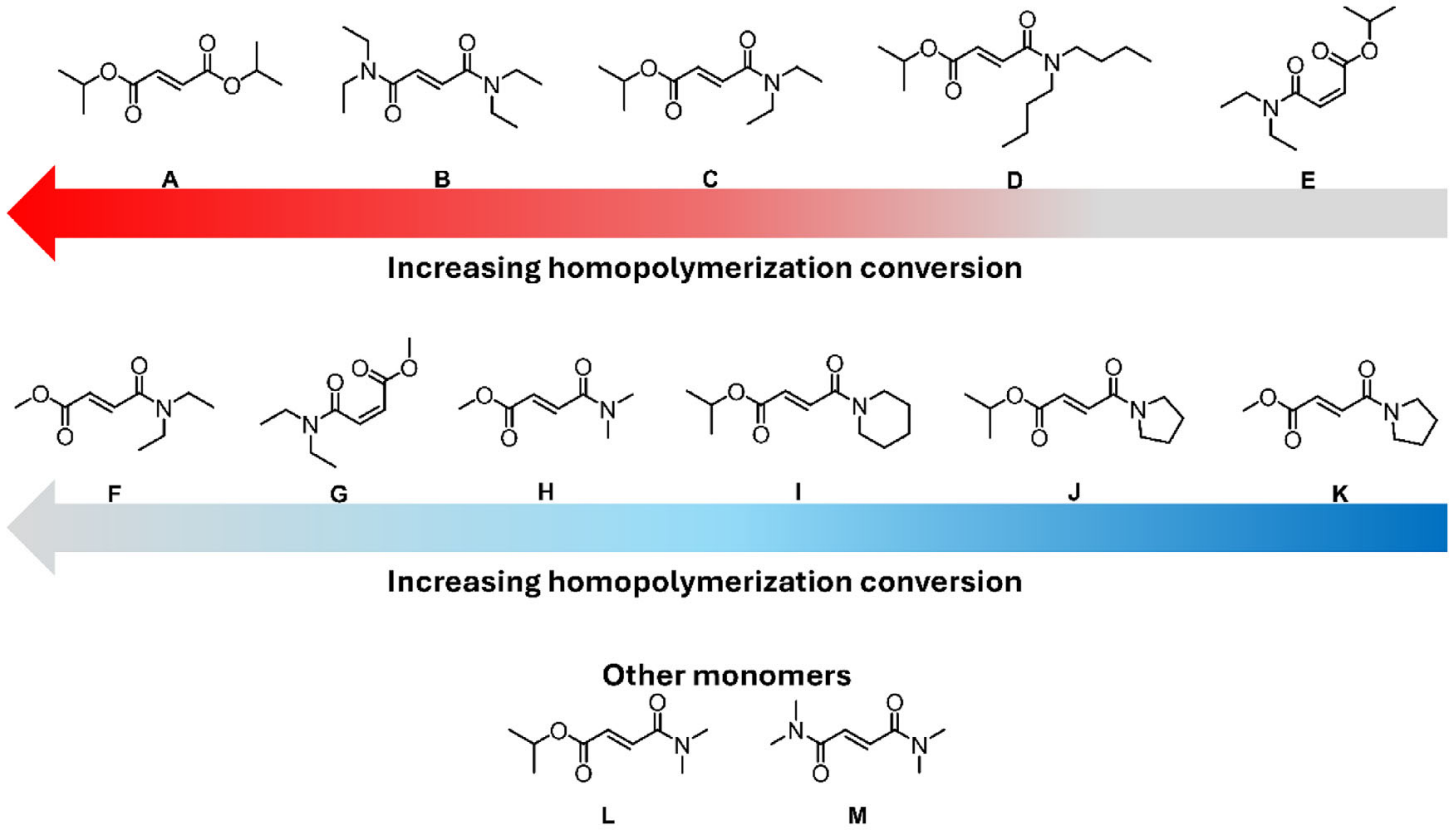
Monomer library investigated in the work from Matsumoto et al. [219]. Proceeding down the alphabet, monomers have lower conversion during conventional radical homopolymerization.

**FIGURE 17:**
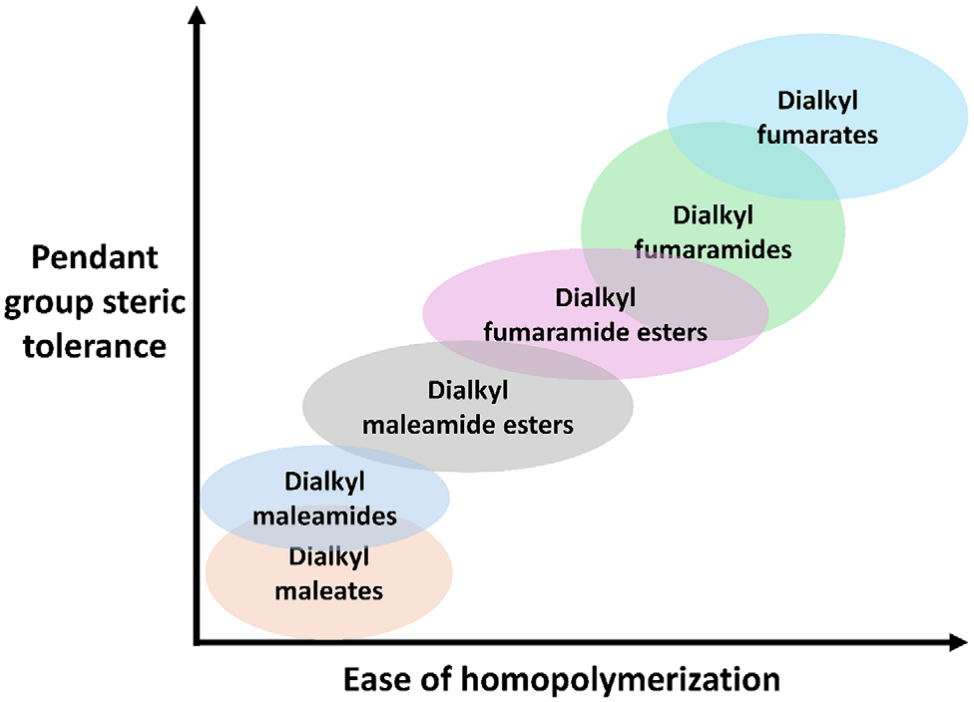
Cartoon representation of pendant‐group size tolerance vs ease of homopolymerization for the monomer classes: dialkyl fumarates, dialkyl fumaramides, dialkyl fumaramide‐esters, dialkyl maleamide‐esters, dialkyl maleamides, and dialkyl maleates. Placements are based on literature trends used as a design guide rather than a quantitative metric.

It is known that maleates do not homopolymerize unless in the presence of an isomerization catalyst; contrarily, maleamide‐esters can homopolymerize [219]. To the best of our knowledge, no studies have been published on the homopolymerization of maleamides. The differences in reactivity among the various monomer classes may stem from the variation in electron density depending on the substituents, i.e., amide vs ester functionality. A study by Matsumoto et al. [219] highlights the difference in reactivity of the two published monomer classes (Figure 16) [219]. A range of fumaramide‐esters (**F** and **C**, DAFAE, Figure 16) and maleamide‐esters (**G** and **E**, DAMAE, Figure 16) were homopolymerized between 60°C and 120°C in bulk. In all instances, the respective DAFAE comonomers reached higher conversions. For both the DAMAE and DAFAE derivatives, the bulkier substituents resulted in higher monomer conversions, as noted with maleates and fumarates; a larger substituent limits the rate of bimolecular termination [219]. The study by Matsumoto et al. [219] thereafter compares several MAnh class derivatives (fumaramide‐ester, **C** and **F** (Figure 16); fumarate, **A** (Figure 16); and fumaramide, **B** (Figure 16)), noting that fumarates and fumaramides reached significantly higher monomer conversions than fumaramide‐esters (reactivity order of **A** > **B** >> **C** > **F**, Figures 16 and 17) during a homopolymerization. In the work by Matsumoto, a monomer library (Figure 16A–C,E,L,M) was copolymerized with styrene (bulk, 60°C) [219], where DAMAE comonomers have significantly lower reactivity in the copolymerizations with styrene in comparison to DAFAEs, and incorporation of either comonomer decreased as substituent size increased [219]. Notably, monomers consisting of cyclic‐amine (Figure 16I–K) and methylamine (Figure 16H) were shown to give poor conversions (< 10%) and generate polymers with molecular weights of less than 1000 g/mol. This was hypothesized to be a result of degradative chain transfer events taking place via the *N*‐alkyl group [219].

**FIGURE 18:**
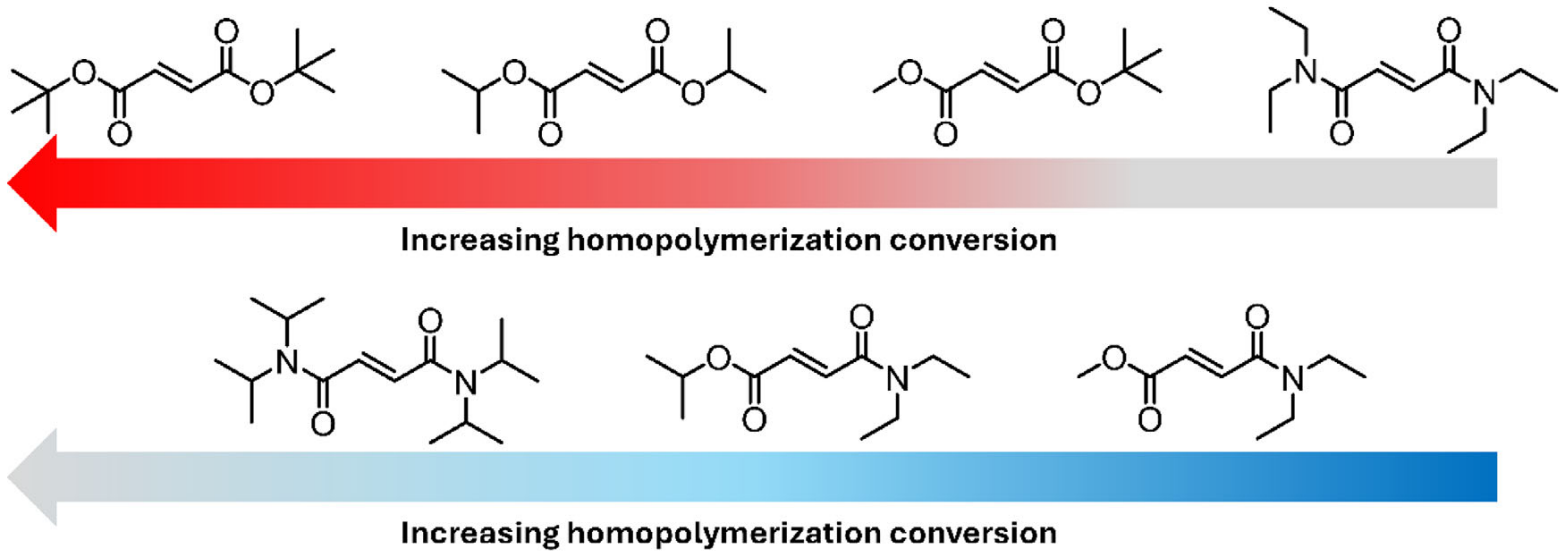
Library fumarates, fumaramide, and fumaramide‐esters from the work by Otsu et al. [220].

A study by Otsu et al., in addition, observed the same homopolymerization reactivity trend noted by Matsumoto et al. [220], i.e., higher homopolymerization conversions were observed for fumarates > fumaramide > fumaramide‐ester (Figures 17 and 18). The work by Otsu et al., in addition, highlights that larger substituents limit bimolecular termination in homopolymerization and, therefore, increase monomer conversion. Fumaramides were emphasised to have a pendant group size upper boundary; too large a substituent afforded limited monomer propagation [220]. The substituent size dependency is less apparent in fumarates due to fewer substituents on the heteroatom functionality (esters being singly substituted and amides doubly substituted) (Figure 18).

**FIGURE 19:**
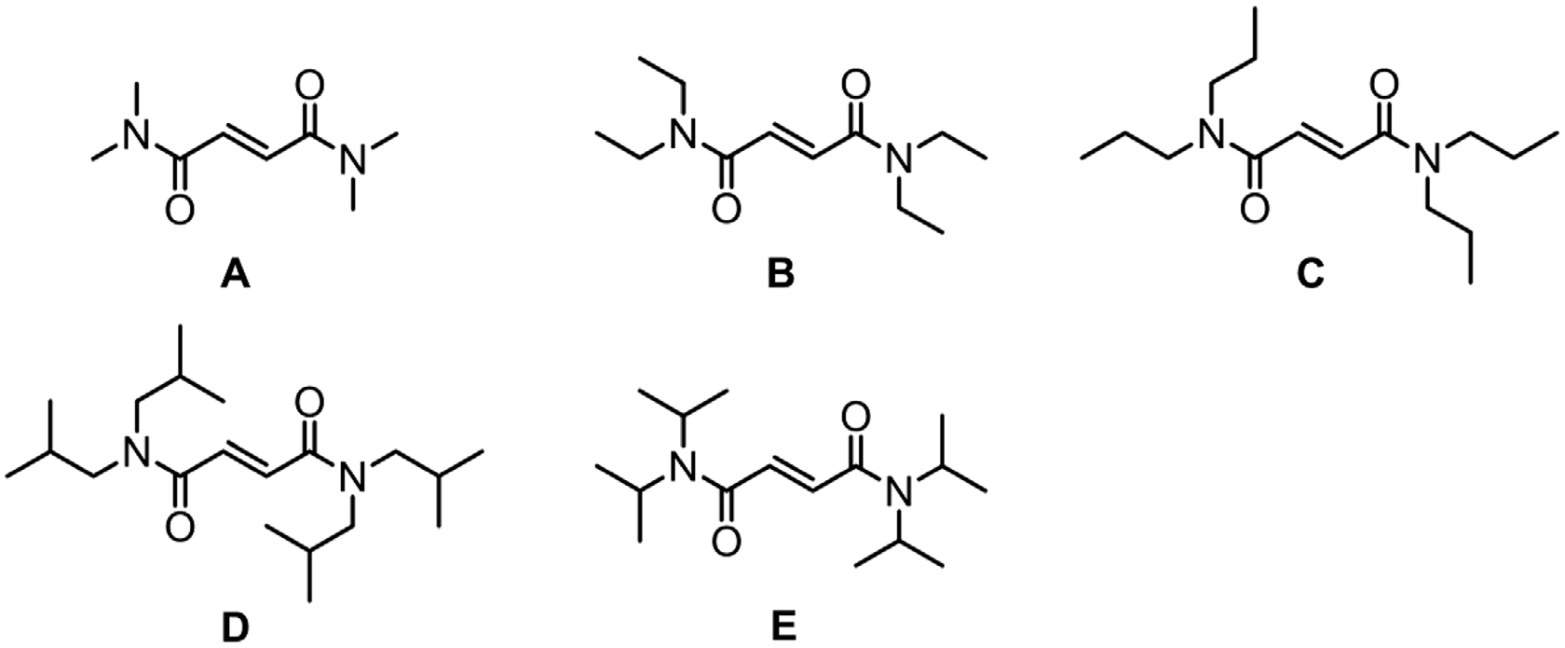
N, N’‐dialkyl fumaramide monomers investigated by Matsumoto et al. [221].

For dialkyl fumaramides (DAFAs), it is understood that the homopolymerizations are dependent on the size of the pendant amine groups, more so than for the ester‐amide derivatives. A study by Matsumoto et al. focused solely on dialkyl fumaramides with secondary amines (Figure 19) [221]. The substituent bulk had a significant effect on the rate of homopolymerization; the larger pendant groups attained the lower monomer conversion: **A** > **B** > **C** > **D** > **E** (Figure 19). Structurally, amine substituents are doubly substituted (oxygen atoms contain a single substituent), and therefore bimolecular termination events (fumaramides and fumarates) are restricted as the pendant group size increases; however, propagation can also be restricted to differing degrees for fumaramides (and to a lesser extent in fumarates). To date, there are no CRP procedures for diamide and amide‐ester fumaramides and maleamides, which presents itself as an opportunity for the research community.

Dialkyl maleamides (DAMAs), dialkyl maleamide‐esters (DAMAEs), dialkyl fumaramides (DAFAs), and dialkyl fumaramide‐esters (DAFAEs) are important, but underexplored classes of MAnh derivatives. Across all monomer classes, it is noted that substituent size governs both propagation and termination kinetics. However, challenges such as degradative chain transfer in certain alkylated systems, insufficient substituent‐reactivity studies, and limited CRP investigations remain significant barriers in their widespread adoption by the polymer community. Studies focused on these efforts would significantly expand their utility in modern polymer science.

## 8 Alkyl Maleamic Acid (AMA), Alkyl Fumaramic Acid (AFA), Mono‐Alkyl Maleate (MAM), and Mono‐Alkyl Fumarate (MAF)

Currently, these monomer classes have not been utilized in any radical‐mediated (co)polymerizations. A worthwhile direction would be to investigate monomer activity as a function of the protonation of the carboxylic acid moiety. As observed with maleic, fumaric, and acrylic acids, it is hypothesized that the monomer activity would vary based on the charged state. Although the asymmetrical structure of these monomers may present experimental challenges, they would be well‐suited for aqueous polymerizations. Furthermore, it is hypothesized that the charged state of the carboxylic acid could have implications for the propagating radical carbon atom identity during polymerization. Incorporation of the amide unit into the monomer structure could provide stability within the polymer backbone, potentially resulting in rigid polymers capable of inter‐ and intra‐chain hydrogen bonding interactions. Importantly, there remains room for structural optimization of these monomers by varying the size of the substituents to simultaneously tune steric effects and possibly modulate the charge state of the carboxylic acid functionality.

**FIGURE 20:**
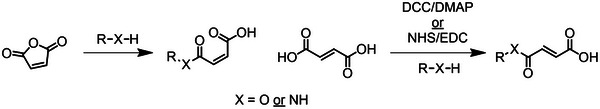
Possible synthetic route to afford alkyl maleamic acid (AMA), alkyl fumaramic acid (AFA), mono‐alkyl maleate (MAM), and mono‐alkyl fumarate (MAF) monomers.

To access maleamic acids without isomerization, we recommend a method developed by Sanchez and coworkers [217]. MAnh is ring‐opened with one equivalent of the desired amine or alcohol (Figure 20) [217]. The fumaramic acid derivatives could be synthesized by coupling reactions with one equivalent of amine or alcohol to fumaric acid via EDC/NHS coupling.

## 9 Future Perspectives

Although research efforts have been conducted within the realm of MAnh and its derivatives, there remain many unanswered questions worth investigating, such as the determination of the impact of the terminal monomer identity in copolymers containing MAnh (other than SMAnh) [82]. It would be worthwhile to determine if the terminal monomer has an impact on chain extension and on chain end functionalization kinetics with other comonomer pairs other than SMAnh. Since the first copolymerizations of styrene and MAnh, it has been noted that it is not possible to conduct copper‐mediated ATRP on MAnh‐containing copolymers. We therefore suggest that the community investigates metal‐free ATRP as an alternative method, which may result in the successful copolymerization of MAnh via ATRP. Maleimides have been primarily copolymerized with styrene; it would be a great opportunity to investigate other, more hydrophilic donor molecules, such as vinyl ethers or *N*‐vinylpyrrolidone, in CRP methods like NMP and ATRP. Dialkyl maleates (DAMs) and Dialkyl fumarates (DAFs), on the other hand, are not compatible with ATRP due to the accumulation of Cu(II). We therefore give the same recommendation as for MAnh: that the community investigates the metal‐free‐ATRP (co)polymerization of these monomers to enable the implementation of this CRP technique with these monomer classes. It is still unknown why DAMs do not homopolymerize without an isomerization catalyst, and therefore, it would be a worthwhile kinetic investigation in the future. There is currently little known about the mechanical properties of the (co)polymers discussed in this review. It would therefore be ideal for further studies to not only focus on the kinetics and/or synthesis of these (co)polymers, but also to compare the mechanical properties of these polymers to more well‐known polymers such as polymethacrylates, etc. A final urgent research direction is the investigation of alkyl maleamic acid (AMA), alkyl fumaramic acid (AFA), mono‐alkyl maleate (MAM), and mono‐alkyl fumarate (MAF) monomers in all types of homopolymerizations and copolymerizations with either hydrophobic or hydrophilic donor monomers such as styrene, alkyl vinyl ether, *N*‐vinylpyrrolidone etc. as there is currently no information that exists on these monomers, their capabilities, and the mechanical properties of the resulting polymers.

## 10 Conclusion

This review highlights the dynamic field of MAnh and its derivatives within the area of radical (co)polymerization (Table 1). Despite significant research conducted that focuses on these monomers, there remains a need for further fundamental research to understand each monomer class. A more comprehensive understanding of monomer class kinetics and reactivities may increase the more general adoption of these underutilized monomers. We hope that this review adds more tools to the polymer chemist's toolbox and, thereby, allows polymer scientists to synthesize novel polymers with enhanced control to design polymer materials with tailored properties.

**TABLE 1:** Summary of the radical (co)polymerizations of MAnh and its derivatives. “Y” = successful studies have been conducted, “N” = no successful studies have been conducted, “?” = has not been investigated. MAnh = maleic anhydride, MI = maleimide, MA = maleic acid, FA = fumaric acid, DAM = dialkylmaleate, DAF = dialkylfumarate, DAMA = dialkylmaleamide, DAFA = dialkylfumaramide, DAMAE = dialkyl maleamide‐ester, DAFAE = dialkyl fumaramide‐ester, MAM = mono‐alkyl maleate, MAF = mono‐alkyl fumarate, AMA = alkyl maleamic acid, AFA = alkyl fumaramic acid.

	Homopolymer	Copolymer	Conventional Radical	ATRP	RAFT	NMP
**MAnh**	**N**	**Y**	**Y**	**N**	**Y**	**Y**
**MI**	**Y**	**Y**	**Y**	**Y**	**Y**	**Y**
**MA**	**N**	**Y**	**Y**	**?**	**Y**	**?**
**FA**	**N**	**Y**	**Y**	**?**	**?**	**?**
**DAM**	**N**	**Y**	**Y**	**?**	**Y**	**?**
**DAF**	**Y**	**Y**	**Y**	**Y**	**Y**	**Y**
**DAMA**	**?**	**?**	**?**	**?**	**?**	**?**
**DAFA**	**Y**	**Y**	**Y**	**?**	**?**	**?**
**DAMAE**	**Y**	**Y**	**Y**	**?**	**?**	**?**
**DAFAE**	**Y**	**Y**	**Y**	**?**	**?**	**?**
**MAM**	**?**	**?**	**?**	**?**	**?**	**?**
**MAF**	**?**	**?**	**?**	**?**	**?**	**?**
**AMA**	**?**	**?**	**?**	**?**	**?**	**?**
**AFA**	**?**	**?**	**?**	**?**	**?**	**?**

## Conflicts of Interest

The authors declare no conflicts of interest.

## Data Availability

The authors have nothing to report.
